# High-content imaging analyses of γH2AX-foci and micronuclei in TK6 cells elucidated genotoxicity of chemicals and their clastogenic/aneugenic mode of action

**DOI:** 10.1186/s41021-019-0117-8

**Published:** 2019-02-05

**Authors:** Akira Takeiri, Kaori Matsuzaki, Shigeki Motoyama, Mariko Yano, Asako Harada, Chiaki Katoh, Kenji Tanaka, Masayuki Mishima

**Affiliations:** Fuji Gotemba Research Laboratories, Chugai Pharmaceutical Co., Ltd., 1-135 Komakado, Gotemba, Shizuoka, 412-8513 Japan

**Keywords:** High content, Genotoxicity, Screening, γH2AX, Micronucleus, TK6

## Abstract

**Background:**

The in vitro micronucleus (MN) test is an important component of a genotoxicity test battery that evaluates chemicals. Although the standard method of manually scoring micronucleated (MNed) cells by microscope is a reliable and standard method, it is laborious and time-consuming. A high-throughput assay system for detecting MN cells automatically has long been desired in the fields of pharmaceutical development or environmental risk monitoring. Although the MN test per se cannot clarify whether the mode of MN induction is aneugenic or clastogenic, this clarification may well be made possible by combining the MN test with an evaluation of γH2AX, a sensitive marker of DNA double strand breaks (DSB). In the present study, we aimed to establish a high-content (HC) imaging assay that automatically detects micronuclei (MNi) and simultaneously measures γH2AX foci in human lymphoblastoid TK6 cells.

**Results:**

TK6 cells were fixed on the bottom of each well in 96-well plates hypotonically, which spreads the cells thinly to detach MNi from the primary nuclei. Then, the number of MNi and immunocytochemically-stained γH2AX foci were measured using an imaging analyzer. The system correctly judged 4 non-genotoxins and 13 genotoxins, which included 9 clastogens and 4 aneugens representing various genotoxic mechanisms, such as DNA alkylation, cross-linking, topoisomerase inhibition, and microtubule disruption. Furthermore, all the clastogens induced both γH2AX foci and MNi, while the aneugens induced only MNi, not γH2AX foci; therefore, the HC imaging assay clearly discriminated the aneugens from the clastogens. Additionally, the test system could feasibly analyze cell cycle, to add information about a chemical’s mode of action.

**Conclusions:**

A HC imaging assay to detect γH2AX foci and MNi in TK6 cells was established, and the assay provided information on the aneugenic/clastogenic mode of action.

**Electronic supplementary material:**

The online version of this article (10.1186/s41021-019-0117-8) contains supplementary material, which is available to authorized users.

## Introduction

The in vitro micronucleus (MN) test is an important component of a genotoxicity test battery [1]. Although manual scoring by microscope is a reliable and standard method used so far to detect micronucleated (MNed) cells, the method is laborious and time-consuming. In pharmaceutical development, a few promising chemicals need to be efficiently selected from a huge number of candidates in a chemical bank. Moreover, to monitor environmental risk, the genotoxicity of the growing number of chemicals in use should be evaluated efficiently to assess the risk for humans. Therefore, high-throughput assay systems that detect MNed cells automatically have become increasingly desired.

A MN is induced by direct DNA damage (clastogenicity) or by retardation of chromosome segregation (aneugenicity). Revealing the clastogenic or aneugenic mode of action (MoA) is important, especially in pharmaceutical development, which needs to define a threshold of genotoxicity [2]. In general, there is no genotoxicity threshold for clastogens, because direct interaction between a chemical and DNA cannot be ruled out even at very low exposure levels. On the other hand, the target molecules of aneugens are not DNA but components of the mitotic apparatus, such as spindle fibers, in which aneugens interrupt polymerization or depolymerization of tubulins. Therefore, DNA per se is not damaged by aneugens, and abnormal chromosome segregations do not occur at a concentration less than that at which the agent interrupts the functions of the mitotic apparatus. Consequently, if the MoA is taken into account, it is acceptable to set a threshold for aneugens [3]. It is especially important to elucidate an aneugenic MoA at an early stage of pharmaceutical development, because this can avoid eliminating promising candidates on which sufficient safety margins could be set. Moreover, the advance warning of the MoA would be helpful for designing the non-clinical genotoxicity studies used in the late stage of drug development [4]; for instance, using centromeric fluorescence in situ hybridization (FISH) analyses in MN tests under GLP.

Since MN tests alone cannot discriminate aneugens from clastogens, they are usually employed in combination with immunostaining of the kinetochore or FISH analysis of the centromere to discriminate aneugens from clastogens [3, 5]. However, these techniques are not suitable for screening purposes because they are laborious and time consuming.

γH2AX, a phosphorylated form at serine 139 of the histone protein H2AX, was found to be an important component of DNA damage response, especially in sensing and repairing DNA double strand breaks (DSB) [6]. γH2AX is a binding interface of a DSB-repair complex and is amplified to more than 2 M base pairs in the chromatin region around a DSB locus, which then forms a microscopically visible focus. The γH2AX focus is considered to be a surrogate marker for a wide range of DNA damage because γH2AX foci are induced not only by DSB (e.g. by ionizing radiation) but also by DNA single-strand breaks (SSB) after treatment with UV irradiation, topoisomerase inhibitors, and reactive oxygen species [6, 7]. The combination of MN test and γH2AX detection on a high-throughput platform will make it possible to discriminate aneugens from clastogens in the early screening stages because it was reported that aneugens do not form γH2AH foci [2] whereas clastogens do. Furthermore, imaging analysis makes it possible to morphologically discriminate γH2AX foci from pan-nucleic γH2AX staining, which is believed to be induced by non-genotoxic events, such as apoptosis [8, 9]. Thus, imaging analysis of γH2AX foci would be an optimal method to specifically evaluate genotoxic events.

Rodent-derived cell lines such as CHL [10] or CHO [11] have been used to evaluate genotoxicity in imaging-based high-throughput systems. However, the predisposition of these cell lines to induce false-positive results more frequently than human-derived cell lines has been raised as a disadvantage. In contrast, the human lymphoblastoid cell line TK6 is noted for its low rate of false positives [12]. Results of genotoxicity tests in TK6 showed the best correlation with those of human lymphocytes, probably because the cell line retains intact p53 [13]. In spite of this advantage, TK6 cells have not been used for imaging-based high-throughput systems because it is difficult to fix non-adherent cells on the bottom of assay plates for imaging analysis.

In the present study, we have established a high-content (HC) imaging assay that detects micronuclei (MNi) in TK 6 cells automatically and simultaneously measures γH2AX foci. Four non-genotoxins, 9 clastogens, and 4 aneugens were subjected to the assay system to evaluate MNi, γH2AX foci, and cell cycle arrest using an imaging cytometer, In Cell Analyzer 6000. Lastly, the advantages of the test system for evaluating genotoxicity for screening purposes is discussed.

## Materials and methods

### Chemicals

The test chemicals, the venders, and the CAS Nos. are enumerated in Table 1. The non-genotoxins were sodium dodecyl sulfate (SDS), sodium chloride (NaCl), D-mannitol (D-Man), and nalidixic acid (NA). The clastogens were methyl methanesulfonate (MMS), *N*-ethyl-*N*-nitrosourea (ENU), mitomycin C (MMC), cis-diamminedichloroplatinum (II) (cisplatin, CDDP), irinotecan (CPT-11, CPT), etoposide (ETP), benzo[*a*]pyrene (BaP), cyclophosphamide (CP), and 2-Amino-1-methyl-6-phenylimidazo[4,5-*b*]pyridine hydrochloride (PhIP). The aneugens were colchicine (COL), vinblastine sulfate salt (VBS), griseofulvin (GF), and paclitaxel (PT). SDS, MMC, CDDP, CPT, and CP were dissolved in sterile distilled water. NaCl and D-Man were dissolved in the culture medium, RPMI-1640 (Nacalai Tesque, Kyoto, Japan) supplemented with 10% fetal bovine serum (FBS, Invitrogen, Carlsbad, CA, USA), 1 mmol/L of sodium pyruvate (Invitrogen), 100 units/mL of penicillin-streptomycin (Invitrogen) and 10 mmol/L of 4-(2-hydroxyethyl)-1-piperazineethanesulfonic acid (HEPES, Invitrogen). MMS, ENU, ETP, BaP, PhIP, COL, GF, and PT were dissolved in dimethyl sulfoxide (DMSO). NA was suspended in DMSO.

**Table 1:** Test chemicals used in the present study

Class	Chemicals	Abbreviations	CAS No.	Venders	Major mode of genotoxicity
Non-genotoxins	Sodium dodecyl sulfate	SDS	151-21-3	Sigma-Aldrich, St. Louis, MO, USA	-
	Sodium chloride	NaCl	7647-14-5	Wako Pure Chemical Corporation, Osaka, Japan	-
	D-Mannitol	D-Man	69-65-8	Sigma-Aldrich	-
	Nalidixic acid	NA	389-08-2	Sigma-Aldrich	-
Clastogens	Methyl methanesulfonate	MMS	66-27-3	Sigma-Aldrich	Guanine N7 alkylation
	*N*-ethyl*-N*-nitrosourea	ENU	759-73-9	Sigma-Aldrich	Guanine O^6^ alkylation
	Mitomycin C	MMC	50-07-7	Kyowa Hakko Kirin, Tokyo, Japan	Cross-linking
	Cisplatin	CDDP	15663-27-1	Sigma-Aldrich	Cross-linking
	Irinotecan	CPT	100286-90-6	Sawai Pharmaceutical, Osaka, Japan	Topo I inhibitor
	Etoposide	ETP	33419-42-0	Sigma-Aldrich	Topo II inhibitor
	Banzo[*a*]pyrene	BaP	50-32-8	Sigma-Aldrich	Guanine N^2^ alkylation by metabolic activation
	Cyclophosphamide	CP	6055-19-2	Sigma-Aldrich	Guanine N7 alkylation by metabolic activation
	2-Amino-1-methyl-6-phenylimidazo[4,5-*b*]pyridine hydrochloride	PhIP	105650-23-5	Wako Pure Chemical Corporation	Guanine C8 alkylation by metabolic activation
Aneugens	Colchicine	COL	64-86-8	Sigma-Aldrich	Microtubule-disruption
	Vinblastine sulfate salt	VBS	143-67-9	Sigma-Aldrich	Microtubule-disruption
	Griseofulvin	GF	126-07-8	Sigma-Aldrich	Microtubule-disruption
	Paclitaxel	PT	33069-62-4	Sigma-Aldrich	Microtubule-disruption

### Cells

Human lymphoblastoid TK6 cells were obtained from the American Type Culture Collection (ATCC, Manassas, VA, USA). Cells were cultured in the culture medium at 37 °C in a humidified atmosphere with 5% CO_2_. The doubling time of the cells was approximately 14 h and the modal chromosome number was 47. When treatment was for 24 h without metabolic activation of S9 mix, a cell suspension was prepared at 5.3 × 10^4^ cells/mL in fresh culture medium and seeded into 96-well flat-bottomed plates at 8 × 10^3^ cells/well (150 μL/well) immediately before the chemical treatment. When treatment was for 3 h with S9 mix followed by a 21-h recovery period (for MN detection), a cell suspension was prepared in the culture medium supplemented with 22% of S9 mix at the same cell density as described above. When treatment was for 4 h with S9 mix (for γH2AX focus detection), the cell suspension was prepared at 2.13 × 10^5^ cells/mL in the culture medium supplemented with 22% of S9 mix and seeded into 96-well flat-bottomed plates at 3.2 × 10^4^ cells/well (150 μL/well). The S9 mix was prepared by mixing cofactor solution with S9 so that S9 was 12% of the S9 mix solution. The S9 fraction (Wako Pure Chemical Corporation) was derived from the liver of male Sprague-Dawley rats treated with enzyme-inducing agents, phenobarbital and 5, 6-benzoflavone. One milliliter of the cofactor solution consisted of 5.3 mg β-NADP^+^, 2.3 mg glucose-6-phosphate, 0.11 mL of 0.08 mmol/L MgCl_2_, 0.11 mL of 0.6 mol/L sodium phosphate buffer (pH 7.2), and 0.77 mL of the culture medium. After 50 μL of test chemical solution/suspension was added to 150 μL of the cell suspensions in each well, the final concentration of S9 was approximately 2% (22% × 12% × 150/200 μL).

### Treatment with chemicals

Each solution or suspension of the chemical was prepared at a concentration 100-fold higher than the final maximum concentration. The maximum concentrations were selected so that around 50% of relative cell counts (RCC) or relative increase in cell count (RICC) were obtained. Then, the solutions were diluted with culture medium to a concentration 4-fold higher than each final maximum concentration. The resulting solutions/suspensions were serially diluted at a ratio of 1.5 or 1.78 with culture medium to prepare 15 serial concentrations for each chemical. The dilution was conducted in 96-well round-bottomed plates. A 50-μL aliquot of each solution/suspension was added to each well, in which cells had been delivered at the levels of density described in the former section, and mixed by pipetting briefly several times. The cells treated with each chemical were cultured at 37 °C in a humidified atmosphere with 5% CO_2_ for 24 h (without S9 mix), 4 h (with S9 mix to measure γH2AX foci), or 3 h (with S9 mix to detect MNed cells). To detect MNed cells, the medium was removed gently after the 3 h treatment with S9 mix so that approximately 50 μL remained, in order to avoid losing any cells. The cells were washed with 150 μL/well of fresh medium by a gentle pipetting and centrifugation (200×*g* for 5 min at room temperature). After the removal of the medium, 150 μL/well of fresh medium was added and the cells were cultured for 21 h.

### Preparation of fixative

A 4% paraformaldehyde/potassium chloride hypotonic fixative (4% PFA/KCl) was prepared as follows. Eight grams of paraformaldehyde (PFA) was added to 160 mL of ultrapure water that was stirred and heated to 58 °C in a water bath. The PFA was dissolved by adding approximately 80 μL of 1 mol/L NaOH and stirring for up to 30 min at 58 °C. After adding 1.12 g of KCl (final concentration 0.075 mol/L), the solution was cooled on ice and adjusted to pH 7.4 by adding several drops of 1 mol/mL HCl. The volume was adjusted to 200 mL with ultrapure water and stored at 4 °C for up to 2 weeks. The 4% PFA/KCl was diluted with 0.075 mol/L KCl to prepare a 1% PFA/KCl solution immediately before use.

### Fixation of cells on 96-well plates

After the treatment with chemicals, each 96-well plate was centrifuged at 200×*g* for 5 min at room temperature. Most of the culture medium in each well was removed, leaving approximately 50 μL in order not to lose any cells from the aspiration. Then 200 μL of phosphate buffered saline (PBS) was added to each well and the plate was shaken for 10 s. These steps (from the removal of culture medium to the shaking) were conducted automatically with a plate washer (Bio-Washer 405RS, DS Pharma Biomedical, Osaka, Japan) under a programmed protocol. The centrifuge and washing was repeated 3 times. Then the cell suspension was transferred to a 96-well imaging plate (Corning 3842 Poly-D-Lysin Coat, Corning Inc., Corning, NY, USA) and centrifuged at 200×*g* for 5 min at room temperature, with the acceleration and deceleration rate set at minimum.

After removing all besides 50 μL of the supernatant in each well, 200 μL/well of 1% PFA/KCl was added gently, and the plate was left for 1 h at room temperature. The fixative was removed in the same manner as in the former step, and 200 μL/well of PBS containing 10% FBS was added and the plates were left for 15 min at room temperature (this FBS treatment step is not essential). After removing the supernatant in the same manner as in the former step, 200 μL/well of 50% methanol was added and it was left for 15 min at room temperature. Then all the supernatant was removed, 200 μL/well of 50% methanol was added again, and the plate was left at room temperature for 20 min. After removing all of the supernatant, 200 μL/well of 0.05 mol/L sucrose was added to avoid cell aggregation in the following cell-drying step. Then the supernatant was removed, leaving approximately 50 μL of the solution, and then the solution was removed completely with an aspirator equipped with an 8-channel adaptor. The plate was completely dried by blowing with a hair-drier at room temperature (Additional file 1: Figure S1). After an additional fixation with 150 μL/well of 99.5% methanol for 5 min, all of the methanol was removed and the plate was placed upside down on a paper towel. The plate was dried by blowing with a hair-drier and was stored in a plastic bag at − 30 °C.

To compare the number of MNed cells counted by automatic imaging analysis with that counted manually with a microscope, conventional glass slide specimens were prepared by cytospin centrifugation of MMC-treated TK6 cells. Aliquots of the cell suspensions for the glass slide specimens were used as for the imaging analysis in a 96-well plate specimen by the method described above.

### Immunostaining of γH2AX

The fluorescence immunostaining was conducted automatically using a microplate washer dispenser (EL406, BioTek Instruments, Winooski, VT, USA) under a programmed protocol. The plate was blocked with 1% bovine serum albumin in PBS (BSA/PBS) for 1 h at room temperature. After removal of the solution, 50 μL/well of anti-γH2AX (anti-γH2AX, phospho Ser139, mouse IgG [9F3], Abcam, Cambridge, UK) that had been diluted at 1:2000 with 1% BSA/PBS was added to each well and left for 2 h at room temperature. The plate was washed with PBS 3 times, and 50 μL/well of AlexaFluor488-labeled anti-mouse IgG goat antibody (Invitrogen) that had been diluted at 1:2000 with 1% BSA/PBS was added to each well, and then the plate was placed at room temperature for 2 h. After washing with PBS 3 times, the plate was fixed with 150 μL/well of 99.5% methanol for 5 min at room temperature. The methanol was removed and the air-dried plate was stored at − 30 °C until the time for staining. The plate was stained with 100 μL/well of PBS containing 1.4 μg/mL Hoechst33258 (Sigma) and 0.1 or 0.02 μg/mL CellMask Red (Invitrogen). The plate was stored at 4 °C until imaging.

### Imaging analysis

In Cell Analyzer 6000 (GE Healthcare) was used to capture images and KiNEDx (Peak Robotics, Colorado Springs, CO, USA) was used to dispense plates automatically. Excitation filters used were as follows: a UV filter for primary nuclei or MNi stained with Hoechst33258, a blue filter for γH2AX foci visualized by an AlexaFluor488-labeled antibody, and a green filter for cytoplasm stained with CellMask Red. A 20-magnification objective lens was used to detect MNi and γH2AX foci, and a 4- or 20-magnification lens was also used for counting cell numbers. Up to around 1000 cells per well, a total of 3000 cells in 3 wells, were analyzed at each dose to calculate the incidence of MNed cells and to count the γH2AX foci per cell. Cells with pan-nucleic γH2AX staining, which is mainly caused by apoptosis, were excluded from the calculation. M-phase cells were morphologically distinguished from cells in other cell-cycle phases and excluded from γH2AX analysis, because M-phase cells are known to express γH2AX independently of DNA damage response. Cell counts per image field were measured to calculate the percentage of RCC, which was an indicator of the cytotoxicity of each chemical. By using the doubling time of the TK6 cells, which was 14 h in the facility, the nominal cell count at the start of culturing and the percentage of RICC was calculated as well.

Cells treated with the chemicals for 24 h were classified to each cell-cycle stage by their fluorescent intensity and the shape of the nucleus. Cell-cycle arrest was evaluated at the concentration where no severe cytotoxicity was observed. The cell cycle of cells treated for 3 h or 4 h was not analyzed because the treatment period would have been too short to affect the cell cycle [14, 15].

The chemical was judged as positive in the MN test if the incidence of MNed cells (average of 3 wells) increased to twice or more than that of the concurrent control and if the increase was dose-dependent. When the γH2AX focus count per cell increased to twice or more than that of the concurrent control and the increase was dose-dependent, the chemical was judged as positive for γH2AX induction.

## Results

### Preparation of plate specimens and imaging analysis

**Fig. 1:**
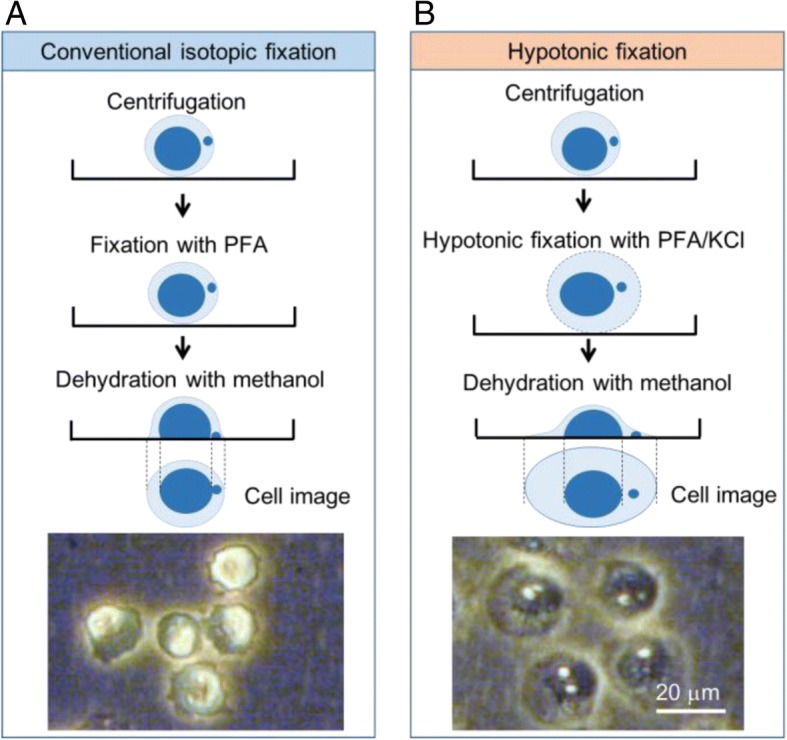
Schema of cell fixation methods and phase-contrast images of TK6 cells on the bottom of a well in the 96-well plate. **a** Fixation by a conventional method using an isotopic condition. Cells in the well were centrifuged and fixed with isotopic paraformaldehyde (PFA). Then the cells were dehydrated with methanol and became attached to the bottom of the well. The method caused cells to shrink through dehydration, and MNi were placed very close to the primary nuclei, which could make it difficult to detect MNi automatically by an imaging algorithm. **b** Fixation by the hypotonic process established in this study. Cells were fixed with hypotonic 1% PFA in 0.075 mol/L KCl (PFA/KCl). The swollen cells spread across the bottom of the well during dehydration. The separation of MNi from the primary nuclei made it easy to detect MNi automatically .

**Fig. 2:**
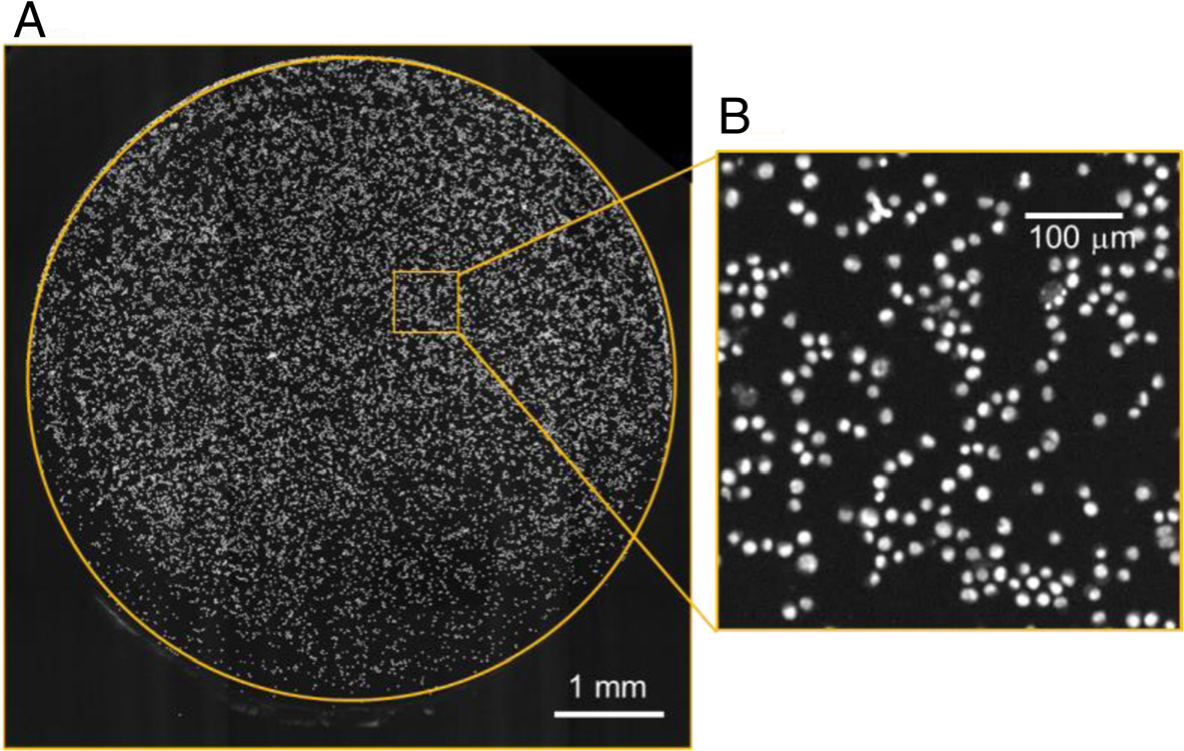
Representative images of TK6 cells attached on the bottom of a 96-well plate. **a** An image of a whole well in the plate and **b** a magnified image of nuclei stained with Hoechst33258. Cells were evenly dispersed and attached on the bottom. The cell binding was tight enough to endure the repeated machine-washings used when immunostaining γH2AX

Fixation of TK6 cells on the bottom of 96-well plates was successfully achieved by the protocol established in this study (Fig. 1). When images of cells fixed with the generally-used isotopic PFA were compared with those fixed hypotonically with PFA/KCl, cell shrinkage was obviously improved. As shown in Fig. 2, the cells were dispersed evenly on the bottom of each well without aggregation or stacking. Furthermore, over 80% of seeded cells became attached to the bottom of wells (data not shown).

**Fig. 3:**
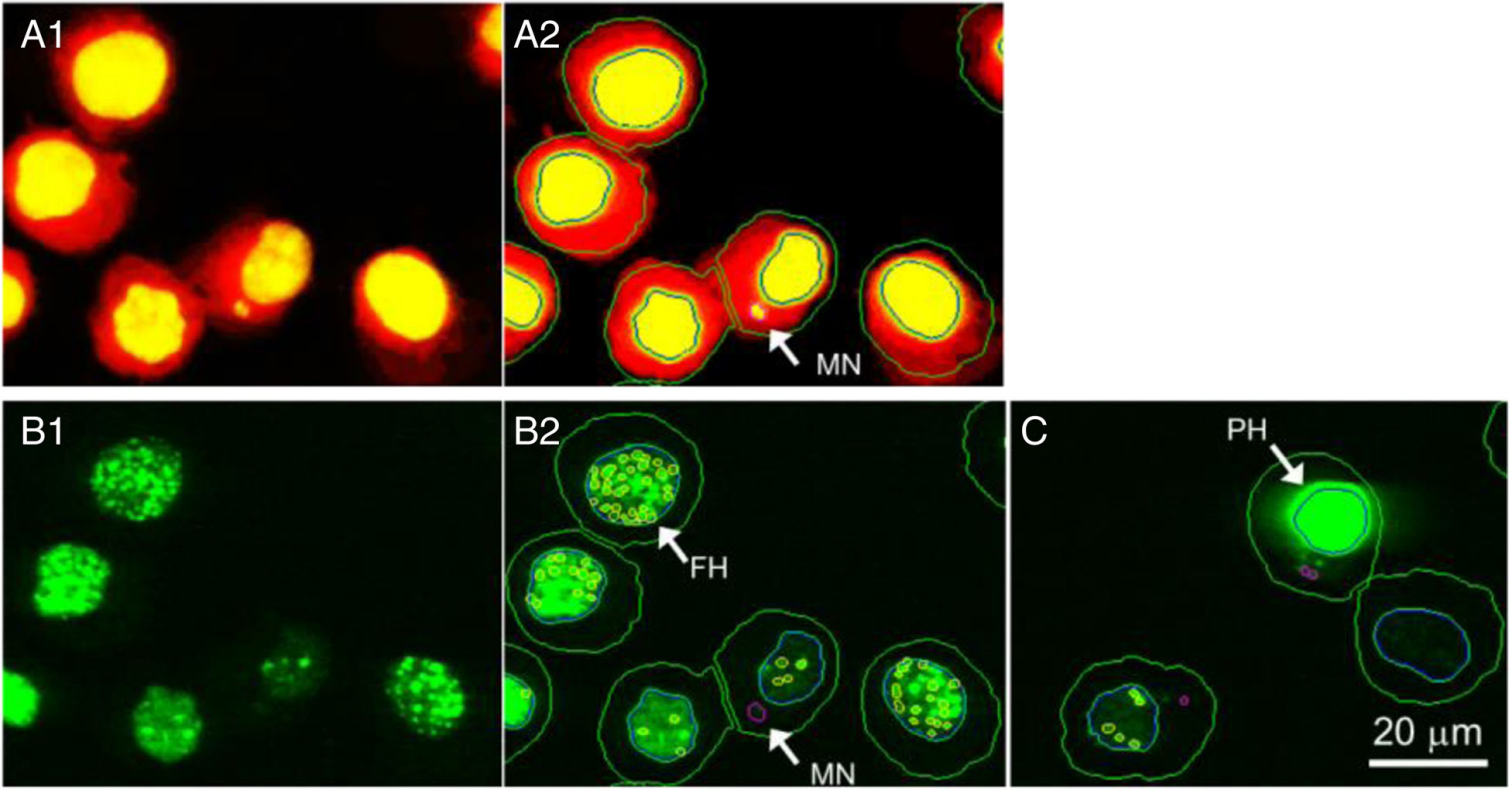
Representative images captured with an imaging cytometer of TK6 cells. Images other than C were obtained at the same time, from the same well. (A1) TK6 cells stained with Hoechst33258 for nuclei and CellMask Red for cytoplasm. The original monochrome images were given pseudo colors, yellow for nuclei and red for cytoplasm. (A2) The arrow shows a MN detected by automatic imaging analysis. (B1) γH2AX foci stained immunocytometrically with AlexaFluor 488-labeled antibody. (B2) Foci of γH2AX (FH) were detected by imaging-analysis. The γH2AX foci and MNi were simultaneously detected by the common protocol of imaging analysis. (C) Pan-nuclear staining of γH2AX (PH), which would be induced in apoptotic cells, was excluded from the analyses

Typical examples from the imaging analyses are shown in Fig. 3 and Additional file 1: Figures S2-S5. When conditions were without S9 mix, both MNi and γH2AX foci were analyzed simultaneously using the common image sources (when S9 mix was used, the images taken to detect MNi and γH2AX foci were different because the treatment regimen was different). MNi could be detected as vesicles in the area between the primary nucleus and the outside boundaries of cytoplasm. Foci of γH2AX could be detected as vesicles in the area of the primary nucleus.

### Comparison with manual MN counting

**Fig. 4:**
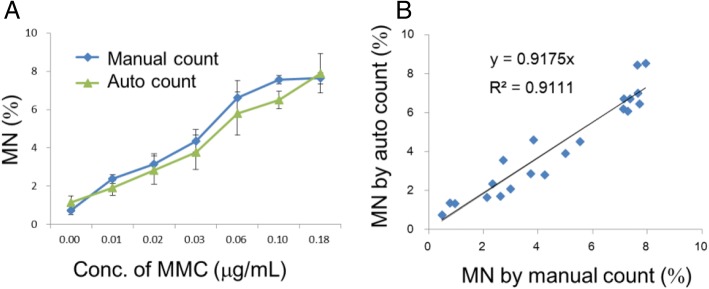
Correlation between scoring MN manually and scoring MN automatically with the imaging-analysis algorithm. MNed TK6 cells treated with MMC were scored manually in specimens on glass slides or automatically using 96-well plates. **a** Percentage of MNed cells increased in a dose-related manner in parallel when scored either manually or automatically. **b** All individual data on MNed cell % were plotted to confirm the correlation between these methods

A high correlation (R^2^ = 0.91) was observed between the frequency of MMC-induced MNed cells measured by the automatic imaging analysis on a 96-well plate and that found by skilled observers manually counting cells on the glass slides by microscope (Fig. 4).

### Measurement of MNed cell frequency and γH2AX focus count

**Fig. 5:**
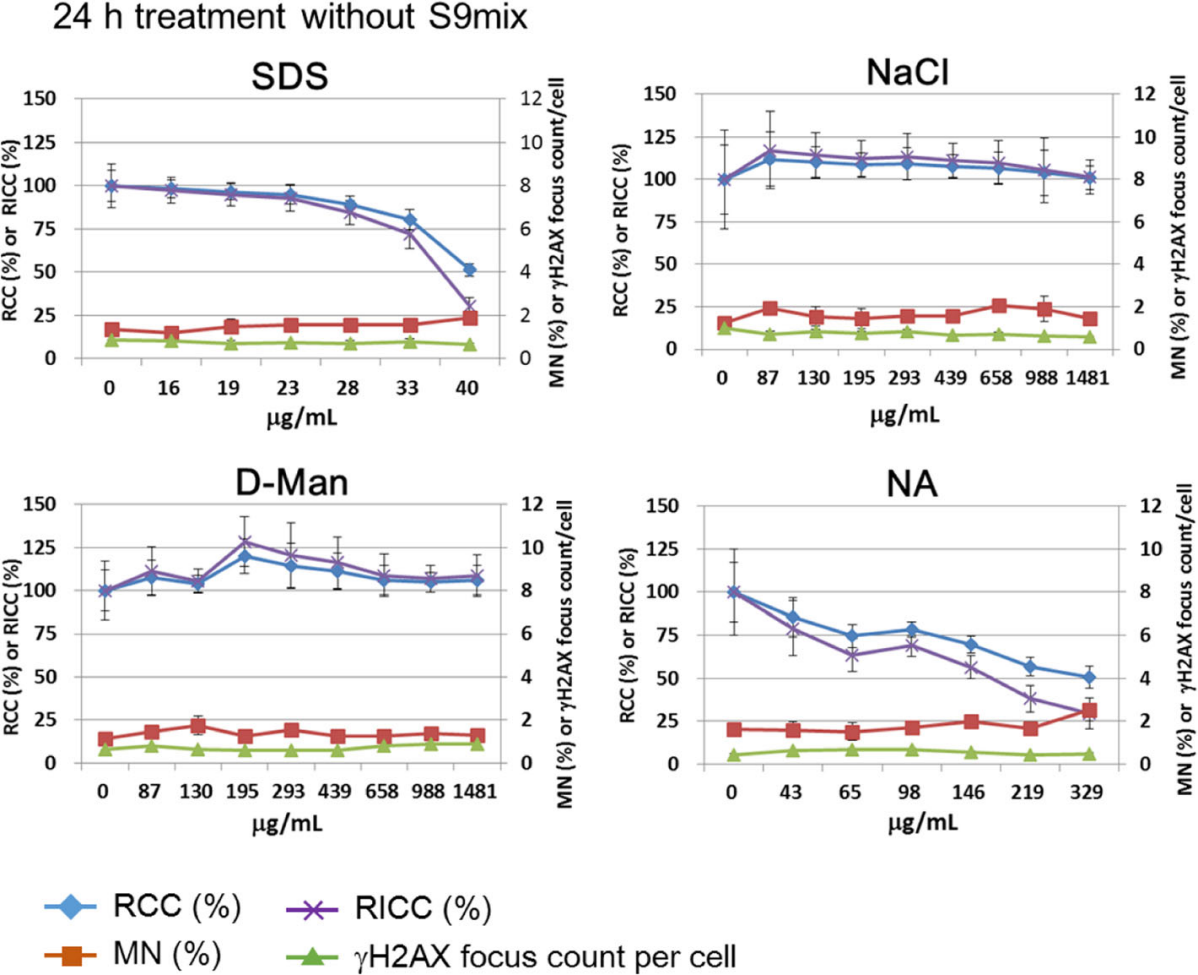
The results of non-genotoxins, sodium dodecyl sulfate (SDS), sodium chloride (NaCl), D-mannitol (D-Man), and nalidixic acid (NA). The blue and purple lines represent relative cell count (RCC) and relative increase of cell count (RICC), respectively. The red and green lines show percentage of MNed cells and γH2AX focus counts per cell, respectively. Non-genotoxins did not induce either MNi or γH2AX foci

**Fig. 6:**
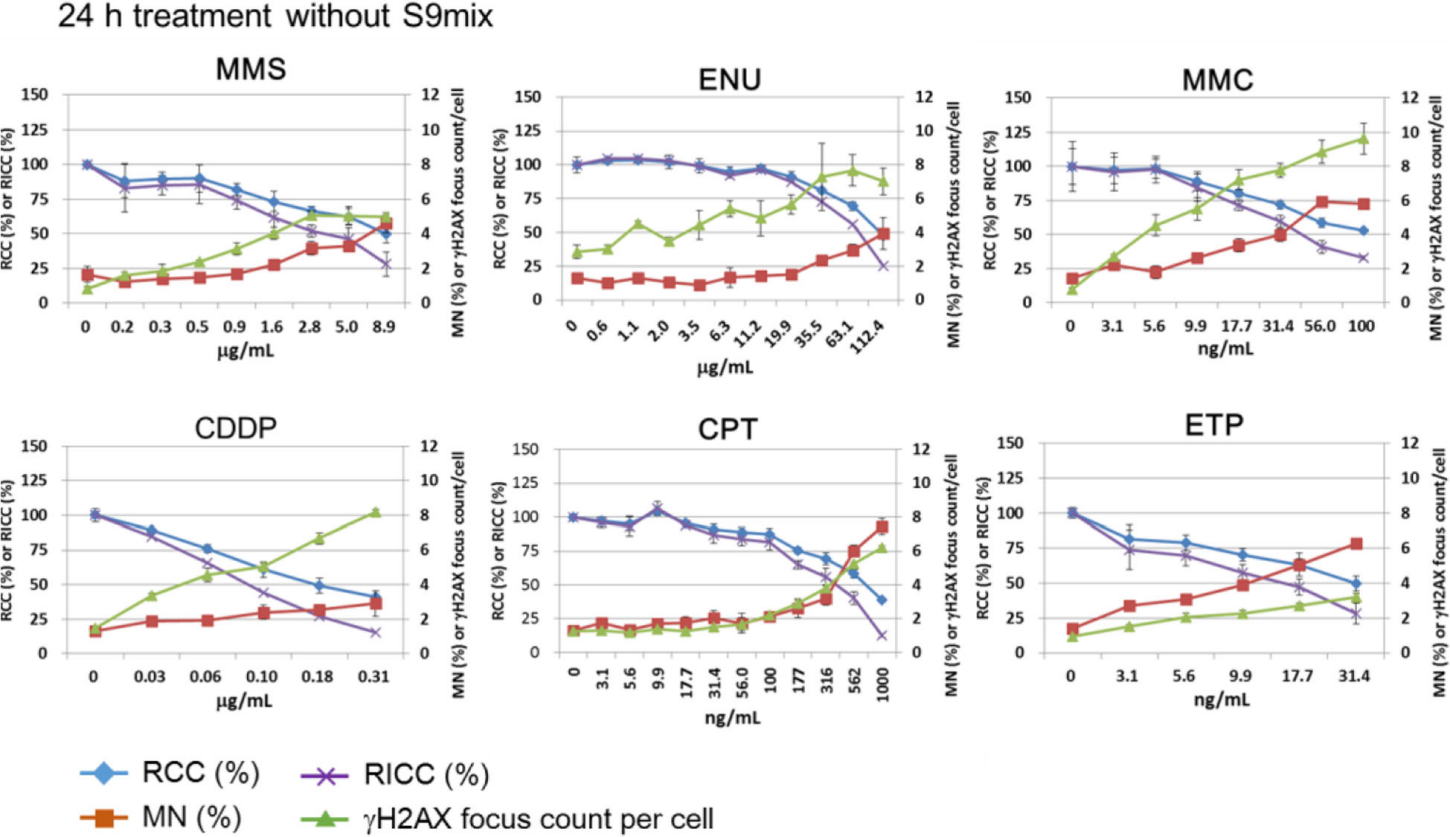
Results for the clastogens that do not need metabolic activation: methyl methanesulfonate (MMS), *N*-ethyl-*N*-nitrosourea (ENU), mitomycin C (MMC), cisplatin (CDDP), irinotecan (CPT), and etoposide (ETP). Both MNed cells and γH2AX foci were increased by all of the clastogens

**Fig. 7:**
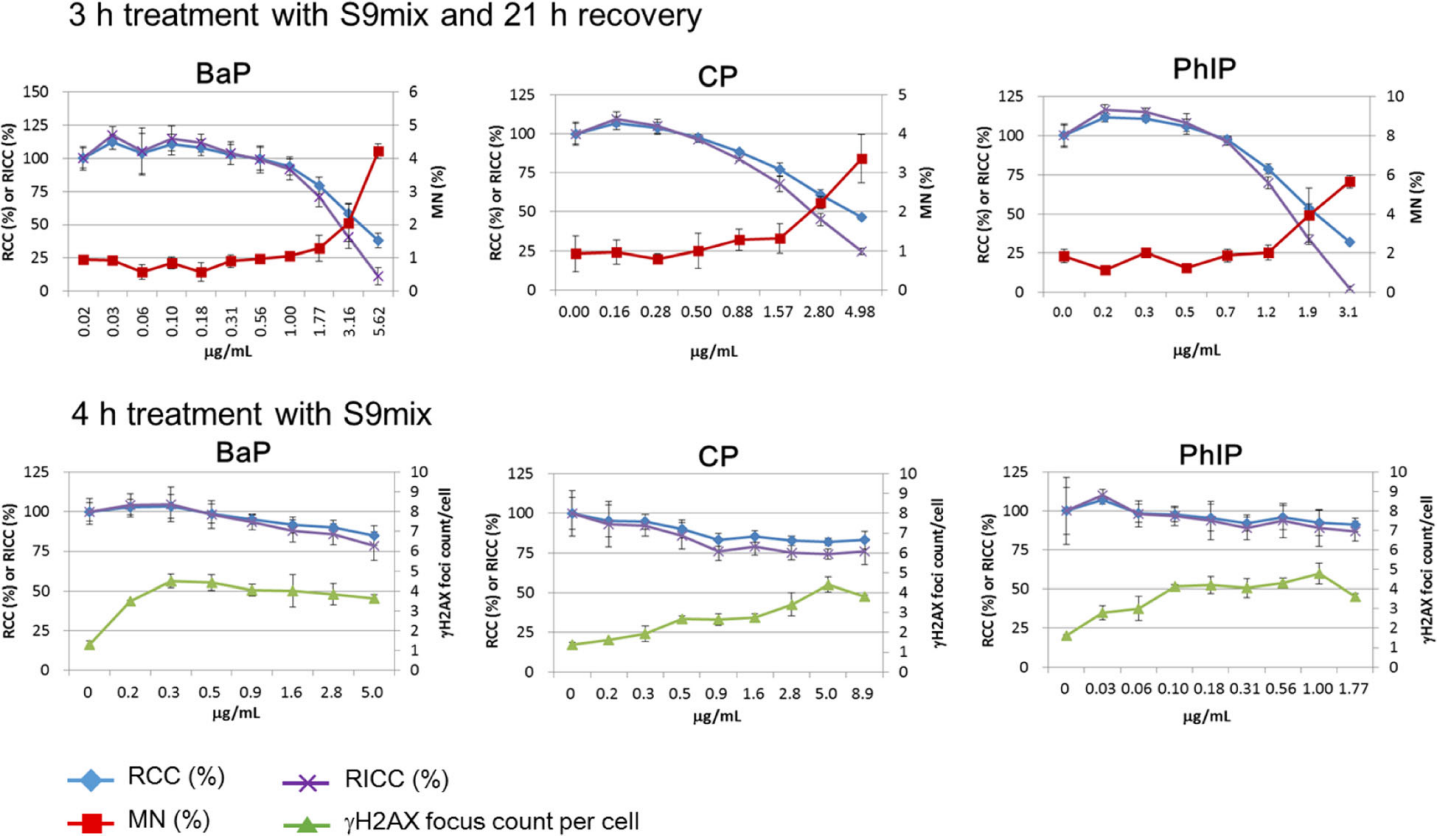
Results for the clastogens that need metabolic activation: benzo[*a*]pyrene (BaP), cyclophosphamide (CP), and 2-amino-1-methyl-6-phenylimidazo[4,5-*b*]pyridine hydrochloride (PhIP). MNed cells were measured after treatment with each chemical and with S9 mix for 3 h followed by recovery culture for 21 h. γH2AX foci were measured after 4 h treatment with each chemical with S9 mix. Both MNed cells and γH2AX foci were increased by all of the clastogens

**Fig. 8:**
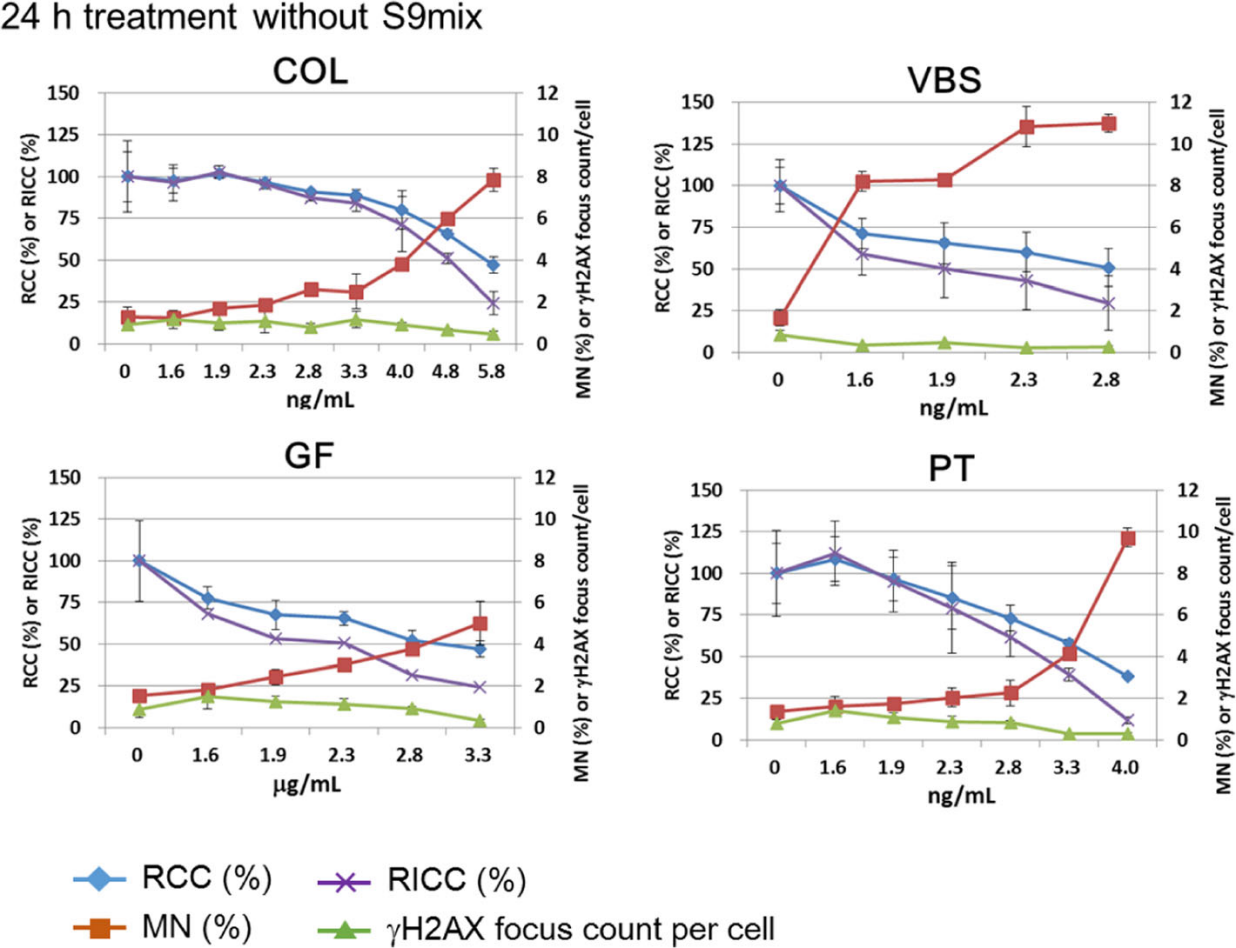
Results for the aneugens colchicine (COL), vinblastine sulfate (VBS), griseofulvin (GF), and paclitaxel (PT). These aneugens induced MNed cells while γH2AX foci were not induced

The results of MNed cell frequency and γH2AX focus count per cell are shown in Figs. 5, 6, 7 and 8. The summary of the results is shown in Table 2. Details of the results of each group, non-genotoxins, clastogens, and aneugens, are described below.

**Table 2:** Summary of the induction of MNi and gH2AX foci

Classs	Chemicals	Results	
		MN induction	gH2AX focus induction
Non-genotoxins	SDS	Neg.	Neg.
	NaCl	Neg.	Neg.
	D-Man	Neg.	Neg.
	NA	Neg.	Neg.
Clastogens	MMS	Pos.	Pos.
	ENU	Pos.	Pos.
	MMC	Pos.	Pos.
	CDDP	Pos.	Pos.
	CPT	Pos.	Pos.
	ETP	Pos.	Pos.
	BaP	Pos.	Pos.
	CP	Pos.	Pos.
	PhIP	Pos.	Pos.
Aneugens	COL	Pos.	Neg.
	VBS	Pos.	Neg.
	GF	Pos.	Neg.
	PT	Pos.	Neg.

### Non-genotoxins

The cells were treated with each non-genotoxin for 24 h without S9 to detect γH2AX foci and MNi. None of the non-genotoxins tested increased the frequency of MNed cells or γH2AX foci (Fig. 5). Treatment with SDS or NA reduced RCC or RICC to 50% or less. Treatment with NaCl or D-Man did not show cytotoxicity even at concentrations higher than 0.5 mg/mL which is the highest dose stipulated for in vitro cytogenesis tests in the ICH S2(R1) guideline. Therefore, all the non-genotoxins tested in this study were judged correctly as negative.

### Clastogens

The cells were treated with each clastogen (MMS, ENU, MMC, CDDP, CPT, and ETP) for 24 h without S9 to detect γH2AX foci and MNi. The cells were also treated with BaP, CP, or PhIP for 4 h with S9 to detect γH2AX foci or for 3 h with S9 followed by a 21 h recovery to detect MNi. All the clastogens tested increased the frequency of both MNed cells and γH2AX foci at the concentrations that reduced RCC up to around 50% (Figs. 6 and 7). Therefore, all the clastogens tested in this study were judged correctly as positive. The induction of γH2AX foci suggested that all the clastogens tested, or their metabolites activated by S9 mix, directly damaged DNA.

### Aneugens

The cells were treated with each aneugen for 24 h without S9 to detect γH2AX foci and MNi. All the aneugens increased MNed cell frequency in the concentration range at which RCC or RICC was reduced to around 50% or less (Fig. 8). Therefore, all the aneugens tested were correctly judged as positive in the MN test. However, none of the aneugens increased γH2AX focus count, even at the concentrations at which RCC or RICC was reduced to 50% or less. These findings suggested that MN induction by the aneugens was not accompanied by direct DNA damage.

### Cell cycle

**Fig. 9:**
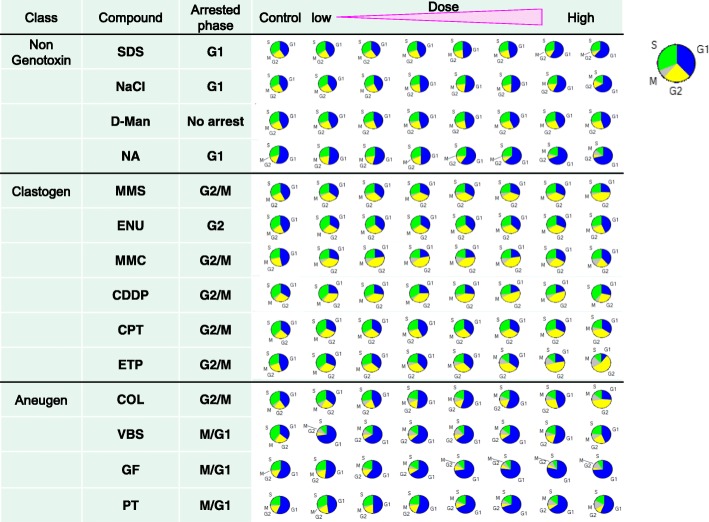
The results of cell-cycle analyses of the chemicals used in the study. The ratio of cells in each cell-cycle phase is shown as a pie-chart that describes the result derived from up to around 1000 cells in a representative well of the 96-well plate. The wells at low to high dose levels are aligned from left to right (the well at the left end represents the concurrent control), the phase at which cell cycle was arrested by treatment with each chemical is also indicated

The result of cell-cycle analysis is presented in Fig. 9. Treatment with D-Man did not show cell-cycle arrest, but cells treated with SDS, NaCl, and NA showed G1 phase arrest, VBS, GF, and PT showed M/G1 arrest, ENU showed G2 arrest, and MMS, MMC, CDDP, CPT, ETP, and COL showed G2/M arrest. COL also increased G1 cells at the mid concentrations.

## Discussion

**Fig. 10:**
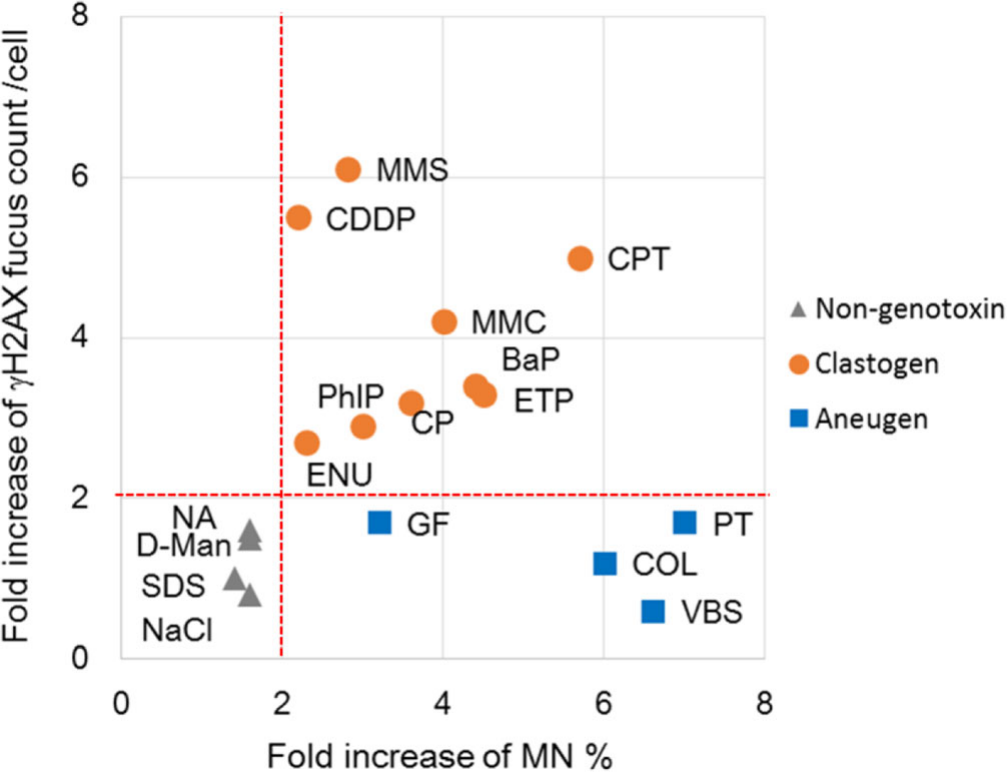
Scatter plots of the chemicals used in the study. The horizontal axis represents fold increase of MN % from each concurrent control level. The vertical axis shows fold increase of γH2AX focus count per cell from each control level. The broken red lines indicate the thresholds for judging positive or negative for MN or γH2AX foci induction, which were 2-fold of control levels. The non-genotoxins are plotted in the lower-left area, which means neither MN nor γH2AX foci was induced. The clastogens are plotted in the upper-right area, while the aneugens are plotted in the lower-right area. This reflects that clastogens induced both MN and γH2AX, but aneugens induced MN without any induction of γH2AX

In the present study, we established a high-throughput genotoxicity test system in which TK6 cells were spread and fixed on the bottom of 96-well plates (Figs. 1 and 2). The system was able to simultaneously analyze the incidence of MNed cells and γH2AX foci (Fig. 3). The incidence of MNed cells detected automatically by the system correlated highly with that of conventional manual scoring by microscope (Fig. 4), and the assay system judged all the non-genotoxins and genotoxins used in the study correctly (Table 2). Therefore, the assay system was suggested to have enough sensitivity and specificity to practically detect the genotoxicity of chemicals. Furthermore, when γH2AX focus evaluation and MNed cell detection were combined, aneugens were clearly discriminated from clastogens. As shown in Fig. 10, when the chemicals used in the study were plotted by the fold-increase of MNed cells and that of γH2AX focus count per cell, the chemicals in each class—non-genotoxin, clastogen, and aneugen—were clearly plotted in their characteristic area. Moreover, cell-cycle analysis was feasible in the assay system (Fig. 9). Taken together, the results suggest that the assay system established in this study is a powerful tool for genotoxicity screening in pharmaceutical development or to identify risk in a large number of industrial or environmental chemicals.

So far, several high-throughput MN detection systems have been reported. Verma et al. reported a system for scoring MN in TK6 cells that combined the flow-cytometric MicroFlow method and the Metafer system, which is based on glass slides [16]. Ogata et al. reported an imaging-based MN scoring test system in CHL/IU cells [10]. Westerink et al. reported an imaging-based MN screening system using CHO-k1 and HepG2 cells [11]. Thougaard et al. presented a validation result of the MicroFlow system for MN detection in TK6 cells [17]. Bryce et al. established a flow-cytometric multiplex test system that detected p53, phospho-H3, and 8 *N* cells to clarify whether the genotoxic mode of action in TK6 cells was aneugenic or clastogenic [18]. The characteristics of these test systems are summarized in Table 3.

**Table 3:** Characteristics of automatic MN detection methods

Methods for MN detection	Advantage	Limitation	Reference
Imaging analysis with slide glass specimens	Easy to confirm the validity of a MN-detection algorithm by means of checking the MN images manually by skilled observers.	Limited to middle-throughput.Difficult to apply multi-endpoint assays.	16
		Difficult to apply robotics for plate preparation.	
Imaging analysis with micro-plates in adherent cells	Easy to confirm the validity of an algorithm by means of checking the MN images manually by skilled observers.	Difficult to apply to non-adherent cells such as TK6 or primary lymphocytes.	10, 11
	Easy to apply robotics for plate preparation.		
Flowcytometric analysis	Easy to apply to non-adherent cells such as TK6 or primary lymphocytes. Applicable to multi-endpoint assay (e.g. gH2AX, H3, p53).	Difficult to confirm whether the MN detected were bona fide.	18
		Difficult to apply robotics for the sample preparation.	
Imaging analysis with micro-plate in non-adherent cells (the method in this article)	Easy to confirm the validity of an algorithm by means of checking the MN images manually by skilled observers.	Difficult to apply robotics for plate preparation.	This article
	Applicable to multi-endpoint assay (e.g. gH2AX).		

Conventional MN tests are based on manual observation through a microscope [19, 20] by skilled observers, who determine whether vesicles in cytoplasm are bona fide MNi. These observers use the information from MN images, such as morphology, size, and roundness, to discriminate bona fide MNi from MN-like vesicles, such as cell debris, fragments of apoptotic nuclei, or auto-fluorescence of chemical precipitates. An advantage of imaging-based automatic MN detection systems is that the standards of MN qualification it applies are in common with the manual method [10, 11, 16]; therefore, the validity of an imaging algorithm used to detect MNi can be easily confirmed by comparing the number of MNi it detects with that detected by skilled observers. Moreover, all images captured by imaging-based methods can generally be stored as digital data, so that re-analysis of the data by a modified algorithm is feasible. On the downside, however, the imaging-based high-throughput assays reported so far can only use adherent cell lines. Though an advantage of the TK6 cell line is the low rate of false positives probably due to intact p53 [13], its use in an imaging-based high-throughput assay is limited because the non-adherent cells are difficult to fix on the bottom of multi-well plates. In fact, the TK6 cell line can be used with the imaging-based Metafer assay [16], but since the assay uses glass-slide specimens, the throughput is only mid-range. Flowcytometry (FCM)-based assays have the advantage of allowing multi-endpoint analysis [18], but it is difficult to confirm whether the MNi detected by FCM are bona fide ones or to re-analyze the data once obtained. In summary, the advantages of using the method established in this study are that it uses the TK6 cell line, which has a low rate of false positives, and it is easy to validate the imaging algorithm.

The use of γH2AX as an endpoint of genotoxicity evaluation has good compatibility with high-throughput platforms because it is induced by a broad range of genotoxic events, such as DSB, SSB, or DNA replication stall, and the foci can easily be specifically detected by fluorescent immunostaining. High-throughput genotoxicity assay systems that use γH2AX as a single endpoint have been reported [21–24]. Furthermore, γH2AX has been used in combination with cellular function as another endpoint. Ando et al. showed that combining γH2AX evaluation with cell-cycle analysis was effective to reveal the genotoxic mode of action [25]. Khoury et al. reported that the combination of γH2AX with phosphorylation of histone H3 at Ser10 was effective to distinguish aneugens from clastogens in 3 cell lines, including HepG2 [26]. In this study, we combined γH2AX with MN detection in TK6 cells and this is the first report of using this combination in an imaging-based high-throughput assay system. The assay system we report here could provide multi-endpoint data, such as MNi, γH2AX, and cell-cycle analysis, from a single 96-well plate specimen. Adding further endpoints is feasible, if appropriate antibodies to detect those endpoints are selected such as phosphorylated histone H3. On the other hand, our method is limited at the present time by the availability of robotics that can prepare the plate specimens, because many experimental steps, including repeated centrifugations and washings, are needed to attach the non-adherent cells to the bottom of the 96-well plate.

The results of MN, γH2AX, and cell-cycle analysis in this report were compared with those in previous reports. MN induction was reported as positive in TK6 cells treated with MMC, COL, VBS, BaP, CP [27], ETP [28], MMS [29], CDDP, and PT [30], but was negative in TK6 cells treated with NA and NaCl [27]. The data contained in our report are consistent with these previous reports in TK6 cells. Furthermore, MN induction by CPT was reported in human peripheral lymphocytes [31] and V79 [32], and by GF in CHL and L5178Y [33]. PhIP was reported to induce MNed V79 cells [34]. D-Man was negative in a MN test using human peripheral lymphocytes [35]. The results in our report are also consistent with these previous reports. With regard to SDS, we could not find any reports of the in vitro MN induction of this compound, so we presume that our negative result for SDS is plausible.

Induction of γH2AX in TK6 cells was previously reported by Bryce et al. [35]. The clastogens they used included MMS, MMC, CDDP, ETP, BaP, and CP, which all induced γH2AX, but the aneugens PT, VBS, COL, and GF and the non-genotoxins SDS, NaCl, and D-Man did not induce γH2AX. γH2AX was induced in human amnion FL cells treated with ENU [36] and in NCI-60 cancer cells treated with CPT [37]. PhIP increased γH2AX foci in V79 cells expressing CYP1A2 and SULT1A1 [38]. No reports on the induction of γH2AX by NA were found. However, NA was reported negative in a GADD45β assay in HepG2 cells [39], an in vitro comet assay, and an in vitro MN test in WTK-1 cells [40]. Therefore, our data is consistent with these previous reports.

Cell cycle was analyzed in TK6 cells in a couple of previous reports. MMS and MMC induced arrest in the G2 or M phase (G2/M) [15] and VBS induced arrest in the M phase [41], and our results are consistent with these previous reports. In our results, ETP induced G2/M phase arrest in TK6 cells. In other reports, TK6 cells were arrested in the S phase immediately after treatment with ETP while in a recovery period, and G2 phase cells increased [41]. Moreover, ETP was reported to induce G2/M phase arrest in 3T3-L1 mouse cells [42]. Therefore, our present finding of G2/M phase arrest in TK6 is thought to be plausible. In A549 cells, CDDP [43] and COL [44] were reported to induce G2/M phase arrest. Although COL mainly induced G2/M arrest, an increase in G1 cells at the mid concentrations was also observed. Nocodazole and vincristine which are microtubule-depolymerizing agents similar to COL were reported to induce mitotic arrest at low concentrations, while G1 and G2 arrest were also induced at higher concentrations in some cell lines [45]. The same mechanisms might underlie the increase in G1 cells observed at the mid concentrations of COL. PT was reported to induce G1 phase arrest in primary fibroblasts of rat or human [46]. NaCl increased the ratio of G1 phase at high concentrations [47]. Therefore our present results are consistent with these previous reports. Although CPT induced G2/M phase arrest in our study, S phase arrest in MCF-7 cells [48] and S phase and G2/M arrest in Caco-2 cells and CW cells have been reported [49]. In our study, GF induced M/G1 arrest, whereas G2/M phase arrest was reported in HL-60 cells [50]. Though, the reason for the discrepancy between our results and these previous reports is unclear, the difference may be related to the cell line used. Though ENU induced G2 arrest in our study, the reagent was reported to induce S phase arrest in neural progenitor cells derived from rat or mouse [51]. In the previous report, the peak of increase in S phase cells was 6 h after the initiation of treatment; however, the cells in our study were treated for 24 h and no S phase arrest was observed. Therefore the discrepancy in cell-cycle arrest may come from the difference in experimental conditions. In our present study, NA and SDS induced G1 arrest, and D-Man did not induce any cell-cycle arrest. However as far as we know, there is no report about cell-cycle arrest induced by these reagents.

## Conclusion

A HC imaging assay to detect γH2AX foci and MNi was established in the TK6 cell line, which is recommended for use in the OECD guideline and is reported to rarely exhibit false-positive results. The assay provided information on the aneugenic/clastogenic mode of action and on cell-cycle arrest. Therefore, the assay is expected to be a useful tool for high-throughput screenings of genotoxicity and its MOA.

## Additional file

Additional file 1: **Figure S1.** A handmade adaptor was used to dry plates with a hair-drier. Pictures A1–A6 explain the method to assemble the adaptor. (A2) Bottoms of a 96-well PCR tube plate were removed with nipper pliers to make small ducts. (A3) A hole the size of the mouth of the hair-drier was cut in an empty box of 96 micro-pipette tips. (A4) The PCR tube plate and the empty box were connected with adhesive tape. (A5, A6) The adaptor was attached to the hair-drier. (B) Each duct of the PCR tube was aligned with each well of the 96-well plates to directly blow the bottom of the well. **Figure S2.** A typical image captured by In Cell analyzer 6000 (GE Healthcare). TK6 cells attached on the bottom of a 96-well plate. The image shows nuclei stained with Hoechst 33258. The Figs. S2 to S5 represent the images obtained from the same cells at the same time. **Figure S3.** Cytoplasm stained with CellMask Red. The image was used to identify the boundaries of the cells. **Figure S4.** Fluorescent immunostaining with anti-γH2AX antibody. **Figure S5.** Imaging analysis by the software ‘Developer’ (GE Healthcare). Light blue and green lines show the boundaries of nuclei and cytoplasm, respectively. Yellow circles represent foci of γH2AX. A MN is shown as a red circle, marked with an arrow labelled MN at center top. M phase cells (M) and apoptotic cells (AP) were excluded from γH2AX foci counting. (DOC 20237 kb)

## Abbreviations

BaP: benzo[*a*]pyrene
BSA: Bovine serum albumin
CDDP: Cisplatin
COL: Colchicine
CP: Cyclophosphamide
CPT: Irinotecan
D-Man: D-mannitol
DMSO: Dimethyl sulfoxide
DSB: DNA double-strand break
ENU: *N*-ethyl-*N*-nitrosourea
ETP: Etoposide
FBS: Fetal bovine serum
FCM: Flowcytometry
GF: Griseofulvin
HC: High-content
KCl: Potassium chloride
MMC: Mitomycin C
MMS: Methyl methanesulfonate
MN: Micronucleus
MNed: Micronucleated
MNi: Micronuclei
MoA: Mode of action
NA: Nalidixic acid
NaCl: Sodium chloride
PBS: Phosphate buffered saline
PFA: Paraformaldehyde
PhIP: 2-amino-1-methyl-6-phenylimidazo[4,5-*b*]pyridine hydrochloride
PT: Paclitaxel
RCC: Relative cell counts
RICC: Relative increase of cell counts
SDS: Sodium dodecyl sulfate
SSB: DNA single-strand break
VBS: Vinblastine sulfate salt
