# Supplementary figures and images for: High-content imaging analyses of γH2AX-foci and micronuclei in TK6 cells elucidated genotoxicity of chemicals and their clastogenic/aneugenic mode of action
Source: Genes Environ. 2019 Feb 5;41:4. doi: 10.1186/s41021-019-0117-8 (PMC6362597; doi:10.1186/s41021-019-0117-8)

Figure S1


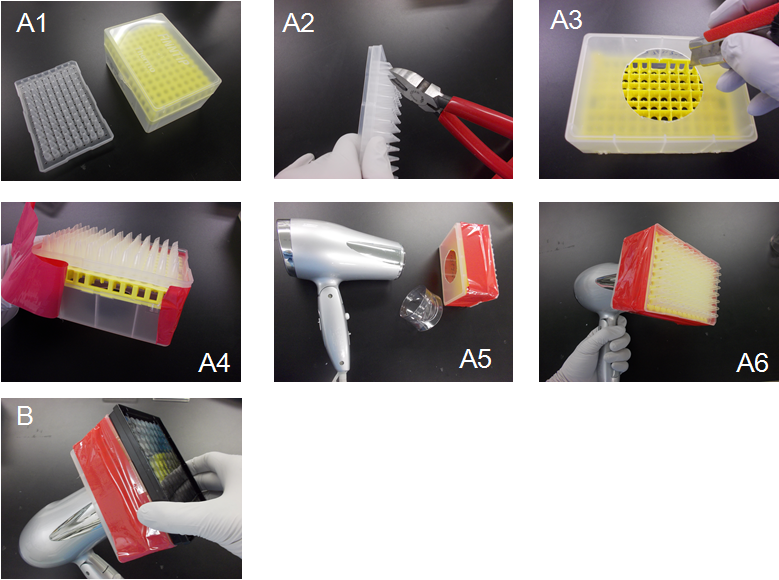


Figure S2


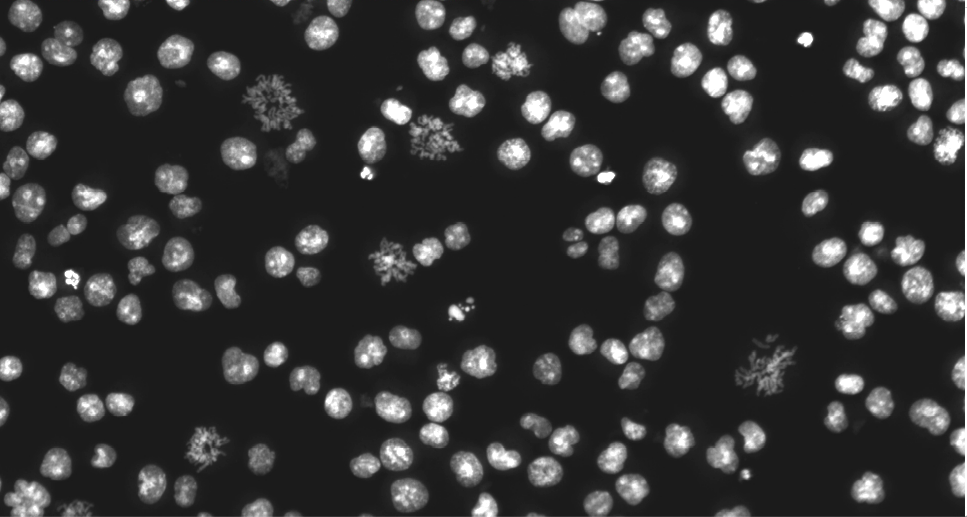


Figure S3


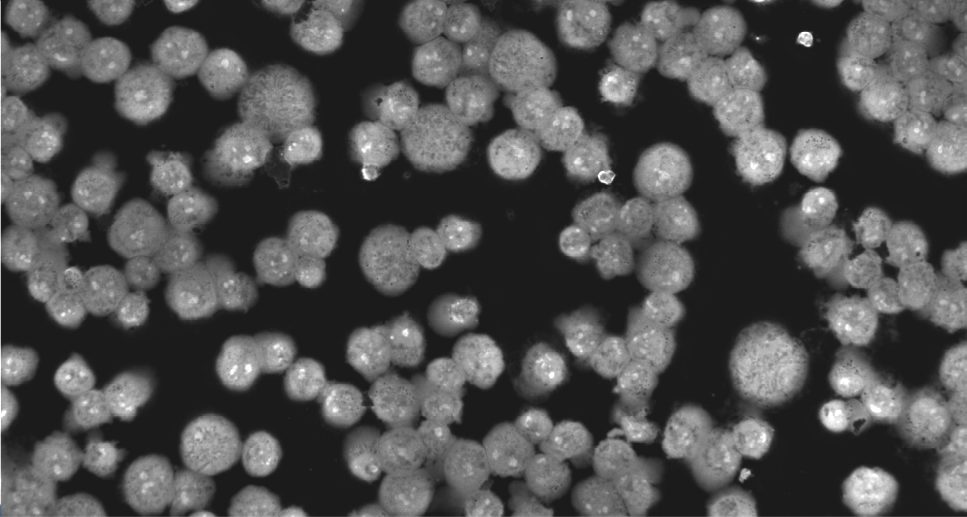


Figure S4


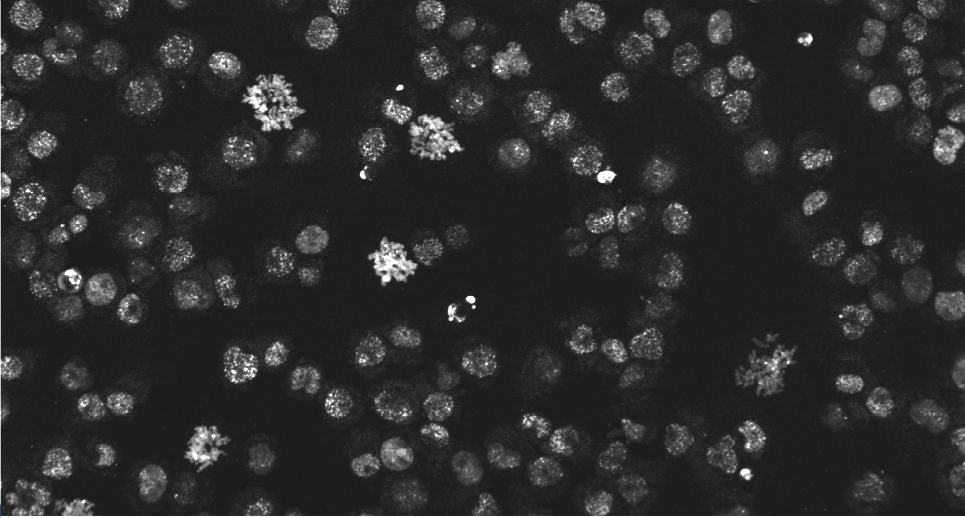


Figure S5


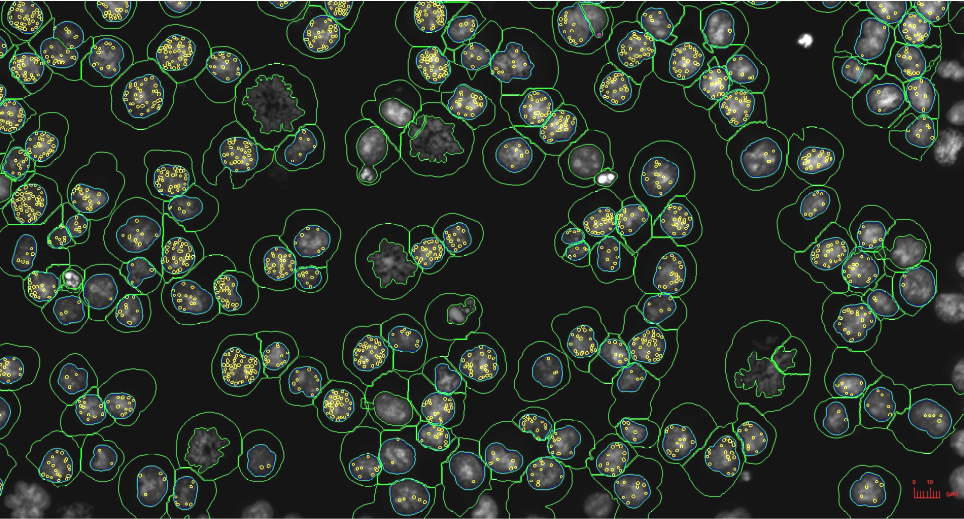


M

M

M

M

M

MN

AP

AP

AP

Supplement: Supplementary file 1 — Figure S1. A handmade adaptor was used to dry plates with a hair-drier. Pictures A1–A6 explain the method to assemble the adaptor. (A2) Bottoms of a 96-well PCR tube plate were removed with nipper pliers to make small ducts. (A3) A hole the size of the mouth of the hair-drier was cut in an empty box of 96 micro-pipette tips. (A4) The PCR tube plate and the empty box were connected with adhesive tape. (A5, A6) The adaptor was attached to the hair-drier. (B) Each duct of the PCR tube was aligned with each well of the 96-well plates to directly blow the bottom of the well. Figure S2. A typical image captured by In Cell analyzer 6000 (GE Healthcare). TK6 cells attached on the bottom of a 96-well plate. The image shows nuclei stained with Hoechst 33258. The Figs. S2 to S5 represent the images obtained from the same cells at the same time. Figure S3. Cytoplasm stained with CellMask Red. The image was used to identify the boundaries of the cells. Figure S4. Fluorescent immunostaining with anti-γH2AX antibody. Figure S5. Imaging analysis by the software ‘Developer’ (GE Healthcare). Light blue and green lines show the boundaries of nuclei and cytoplasm, respectively. Yellow circles represent foci of γH2AX. A MN is shown as a red circle, marked with an arrow labelled MN at center top. M phase cells (M) and apoptotic cells (AP) were excluded from γH2AX foci counting. (DOC 20237 kb) [file 41021_2019_117_MOESM1_ESM.doc]
